# A Rare Presentation of a Complex Mixed Autoimmune Encephalitis Diagnosis: A Case Report and Literature Review

**DOI:** 10.7759/cureus.29607

**Published:** 2022-09-26

**Authors:** Surpreet Khunkhun, Kunal Aggarwal, Humzah Iqbal, Nihal Satyadev, Kamalpreet Mann, Samir Ruxmohan, Gabriela Perez, Hyder Tamton

**Affiliations:** 1 Medicine, University of Medicine and Health Sciences, Basseterre, KNA; 2 Department of Medicine, Icahn School of Medicine Mount Sinai-Elmhurst, New York, USA; 3 Department of Internal Medicine, University of California San Francisco, Fresno, USA; 4 Department of Neurology, Mercy Health, Grand Rapids, Michigan, USA; 5 Department of Neurology, Larkin Community Hospital, Miami, USA; 6 Department of Neurology, Palmetto General Hospital, Hialeah, USA

**Keywords:** plasmapheresis, plasma exchange, paraneoplastic encephalitis, miller fisher, bickerstaff encephalitis, autoimmune encephalitis

## Abstract

This case report presents a unique case of a difficult differential diagnosis of autoimmune encephalitis (AE) in the setting of *Mycoplasma pneumoniae*. A 40-year-old female with a history of Hashimoto thyroiditis, polycystic ovarian syndrome, and a lower respiratory infection presented to the emergency department with new-onset progressive neurological symptoms. These included generalized tonic-clonic seizure and worsening respiratory status that required intubation and tracheostomy. Blood cultures returned positive for *M. pneumoniae*. We concluded this to be a mixed diagnosis case of anti-glutamic acid decarboxylase 65 (anti-GAD65), Bickerstaff’s brainstem encephalitis (BBE), Hashimoto’s encephalopathy (HE), and Miller Fisher Syndrome (MFS) concurrently in the setting of *M. pneumoniae.* Initial treatment with intravenous immunoglobulin showed minimal improvement; however, subsequent treatment with plasmapheresis proved to be beneficial for the patient. Over the course of the plasma exchange therapy (PLEX), the patient slowly became more alert, attentive, and verbal. She was able to answer simple questions and follow commands. Common trends of age, gender, presenting symptoms, associated antibodies, and sessions of PLEX in different AE diseases were identified through a literature review. Only 69.7% of the cases implemented PLEX or plasmapheresis. Currently, there is no standard protocol for the treatment of AE. Our case report aims to present a clinically complicated example of AE and to provide further evidence to support PLEX as an important therapeutic option.

## Introduction

Acute encephalitis is a potentially lethal inflammatory process within the brain that can be caused by infectious agents, toxic metabolites, neurodegenerative dementias, psychiatric diseases, metabolic disorders, vascular disorders, neoplastic disorders, and demyelinating or inflammatory disorders (see Appendix) [1]. Automimmune encephalitis (AE) is a type of encephalitis that targets intracellular and plasma membrane proteins of neurons. Due to the wide variety of clinical manifestations seen in these patients as well as the presence of several subtypes with many overlapping features, the diagnosis of AE can be challenging. They are generally differentiated by the specific autoantibodies present in the patient [2].

Hashimoto’s encephalopathy (HE) involves the presence of anti-thyroperoxidase (anti-TPO) or anti-thyroglobulin antibodies along with myoclonus, hallucinations, seizures, and concurrent thyroid disease [2]. Brain MRI is generally normal in these patients [2]. The diagnosis of anti-glutamic acid decarboxylase 65 (anti-GAD65) antibody-associated encephalitis is made in patients found to have anti-GAD65 antibodies [2]. These patients may present with abnormal postures such as stiff-person syndrome, short-term memory loss, psychiatric symptoms, and seizures [2].

Certain subtypes are associated with an infectious process such as mycoplasma infection leading to autoimmune rhomboid Bickerstaff’s brainstem encephalitis (BBE) [3]. BBE is diagnosed in the presence of anti-GQ1b antibodies along with decreased consciousness, bilateral external ophthalmoplegia, and ataxia [3]. Miller Fisher syndrome (MFS), a variant of Guillain-Barré syndrome (GBS), is another subtype of AE that most commonly arises in a post-infection state. The three most common symptoms are ataxia, areflexia, and ophthalmoplegia [4].

The treatment for AE includes intravenous corticosteroids, intravenous immunoglobulin (IVIG), anti-epileptics, plasmapheresis/therapeutic plasma exchange (TPE), and immunosuppressants. In this article, we document the case of a patient with complex AE following *Mycoplasma pneumoniae* infection, and we aim to address the quality gap between the effectiveness of the various treatment modalities.

## Case presentation

We present a case of a 40-year-old female who arrived at the emergency department (ED) for progressively worsening weakness in all four limbs, overt ataxia, binocular diplopia, and severe dysarthria. Her past medical history was significant for Hashimoto’s thyroiditis, polycystic ovarian syndrome (PCOS)-like features, and remote COVID-19 infection. Collateral information collected from her mother also revealed a family history of rheumatologic disorders.

Upon presentation, her initial vitals were as follows: the temperature was 98.4℉, heart rate (HR) was 86 bpm, respiratory rate (RR) was 23 breaths/minute, BP was 154/92 mmHg, and pulse oximeter reading was 95%. A physical examination revealed spontaneous symmetrical movements in all four limbs, diminished light touch and vibration sensation in all four limbs, intact pain sensation in all four limbs, hyperreflexia in all four limbs, and clonus in the lower extremities, bilaterally. Babinski's sign was positive bilaterally. Furthermore, the patient was nonverbal and was unable to follow any commands. Blood gas showed mild respiratory alkalosis with a pH of 7.47, partial pressure of carbon dioxide of 30.3, partial pressure of oxygen of 81.7, and bicarbonate of 21.7.

Computed tomography (CT) of the brain done with and without contrast revealed several calcified soft tissue scalp lesions but no acute abnormalities. Routine electroencephalograms were suggestive of moderate, diffuse encephalopathy but without epileptiform activity. Given the patient’s presentation along with her history of two infections and Hashimoto’s thyroiditis, there was high suspicion for AE. Thyroid studies revealed high levels (7.54) of thyroid stimulating hormone (TSH), but her levels came down to a normal range during the course of her hospital stay. In order to confirm this suspicion and identify an AE subtype, an extensive antibody screening was conducted (Table 1). The patient was positive for anti-GAD65, anti-TPO, anti-mitochondrial (anti-M2), anti-HLA class I, anti-Ib, anti-IIb/IIIa, and anti-SSA (Ro) antibodies.

**Table 1:** Antibody screening results Anti-GAD65: Anti-glutamic acid decarboxylase; Anti-TPO: Anti-thyroperoxidase; Anti-M2: Anti-mitochondrial; Anti-HLA: Anti-human leukocyte antigen; Anti-SSA: Anti-Sjögren's-syndrome-related antigen A; Anti-CASPR2: Anti-contactin-associated protein-like 2; Anti-NMDAR: Anti-N-methyl-d-aspartate receptor; Anti-VGKC: Anti-voltage-gated potassium channel.

Positive Antibody Results	Negative Antibody Results
Anti-GAD65	Anti-CASPR2
Anti-TPO	Anti-GM1
Anti-M2	Anti-GD1b
Anti-HLA class I	Anti-GQ1b
Anti-Ib	Anti-NMDAR
Anti-IIb/IIIa	Anti-Hu
Anti-SSA	Anti-NMO
	Anti-VGKC
	Anti-Yo

After experiencing a seizure, she was admitted to the neurocritical care intensive care unit (neuro-ICU) and required intubation for airway protection. It is worth mentioning that the patient was found to have *M. pneumoniae* infection a day after admission. In the neuro-ICU, she initially received two rounds of five-day courses of IVIG and steroids. Despite showing initial improvement, she had developed an ictal activity that warranted a five-day course of plasma exchange therapy (PLEX). Following this therapy, the patient showed marked improvement in her level of alertness. However, after multiple failed attempts for ventilation weaning as well as swallowing evaluations, she required tracheostomy placement and percutaneous endoscopic gastrostomy (PEG) tube insertion. Over time, she was able to be weaned off from mechanical ventilation, and she was able to tolerate food without choking.

She was discharged on hospital day 44. At the time of discharge, she was alert and oriented to person, place, and time; she was able to follow commands, and her dysarthria was markedly improved. Treatment post-discharge included chronic immunosuppressant therapy with rituximab, which was to be administered once every three months for two years. Follow-up referrals with pulmonology and gastroenterology were suggested for one to two weeks post-discharge for further management of the tracheostomy and percutaneous endoscopic gastrostomy (PEG) tube, respectively.

## Discussion

**Figure 1:**
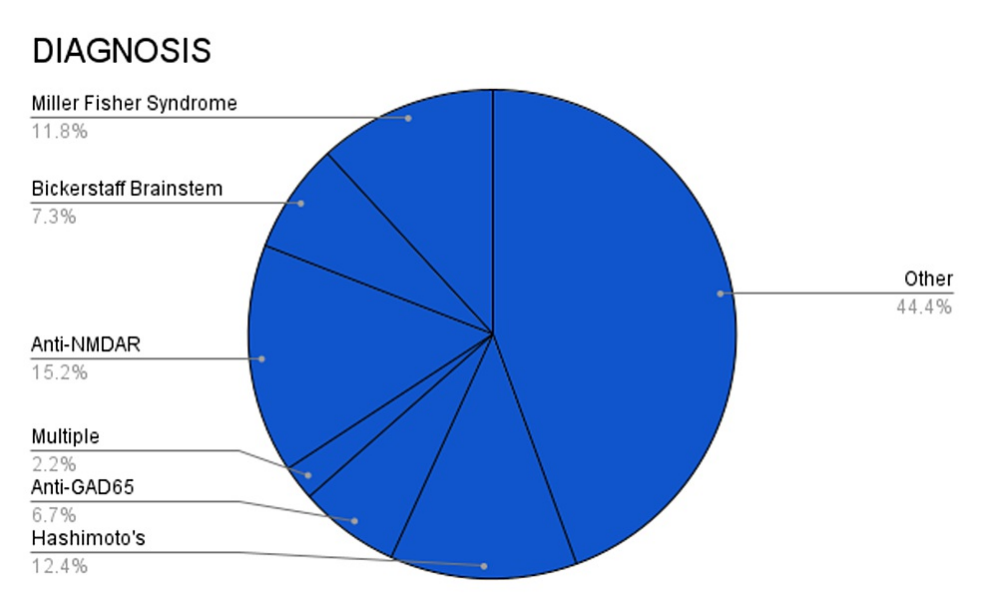
Percentage breakdown of different autoimmune encephalitis subtypes This pie graph summarizes the percentage breakdown of the different autoimmune encephalitis cases identified in our literature review search. Anti-GAD65: Anti-glutamic acid decarboxylase; Anti-NMDAR: Anti-N-methyl-d-aspartate receptor.

A PubMed search was conducted using the keywords “autoimmune encephalitis” OR “bickerstaff encephalitis” OR “miller fisher” OR (“paraneoplastic encephalitis” AND “plasma exchange”) and were screened for case reports in English. A total of 178 cases were identified between the years 1981 and 2020. Figure 1 shows the percentage breakdown of the different AE subtypes identified in our literature review search. Of the cases that reported gender (n = 176), 71 (40.3%) were males and 105 (59.7%) were females. Of the cases that reported age (n = 176), 48 (27.2%) were eight years old or younger and 128 (72.7%) were 19 years old or older. Age of onset ranged from one to 79, with a median age of 34.4 years.

**Figure 2:**
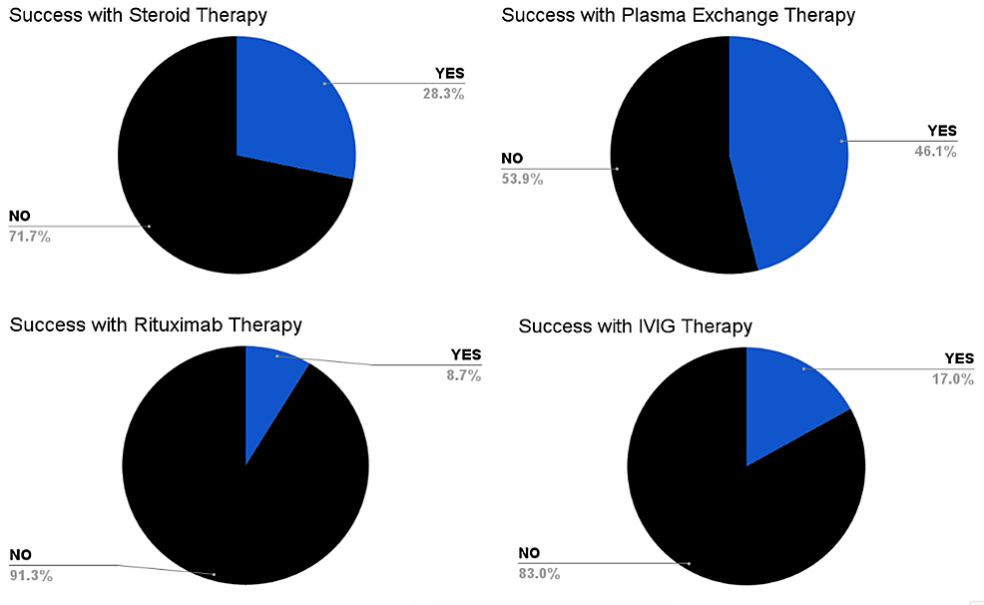
Percentage breakdown of successful therapy with different treatment options The pie graphs in this figure show the percentage breakdown of the success of different treatment options discovered in our literature review search regarding AE. AE: Autoimmune encephalitis.

PLEX or plasmapheresis therapy was implemented in 124 cases. From the cases that reported the number of rounds of PLEX or plasmapheresis therapy (n = 104), the average number of rounds was 6.3. Approximately, 50 cases (28.0%) reported the presence of residual neurological symptoms after treatment and clinical improvement. Of the cases that reported successful therapy, Figure 2 shows that 38 (21.3%) included IVIG as successful, 47 (26.4%) included corticosteroids, and 108 (60.7%) included PLEX or plasmapheresis.

The literature review also revealed certain side effects and complications following plasmapheresis/PLEX including a hypotensive crisis [5], a right wrist drop with a left foot drop [6], nausea, hypotension [4], and even death [7,8].

A quarter (25%) of the cases that were treated with PLEX still experienced residual symptoms afterward, whereas the overall residual symptom rate in all the different treatment cases was 28%. The residual symptoms list included reduced muscle strength, recurring visual/acoustic hallucinations, dysarthria, retrograde amnesia, anterograde amnesia, mutism, seizures, hearing/speech/motor deficits, hyper/hyporeflexia, myoclonus, absent deep tendon reflexes (DTRs), varying oculomotor deficits (most commonly reporting limited abduction of R eye), and spastic paraparesis. Additional therapies that proved to be effective in treating patients diagnosed with AE conditions include alemtuzumab [9], azathioprine [10], bortezomib [11], cyclophosphamide [12-16], and rituximab [3,8,17-30].

We present a unique and complex case that is suggestive of mixed AE secondary to various etiologies. Our patient presented with ataxia, dysarthria, blurry vision, and generalized weakness, symptoms that are typical of encephalitis. However, the patient’s history and laboratory workup raised the suspicion of AE with overlapping features of four distinct subtypes. Her past history of Hashimoto’s thyroiditis along with positive anti-TPO titers was in alignment with a presentation of HE. Her positive anti-GAD65 titers made a diagnosis of anti-GAD65-associated encephalitis plausible as well. Furthermore, her prior history of *M. pneumonia* and a COVID-19 infection made MFS another possible differential. The association between these two infections and MFS has been well documented in the literature. Generally, a diagnosis of BBE is made when the patient shows decreased level of consciousness, bilateral external ophthalmoplegia, and ataxia with the presence of anti-GQ1b antibodies [2]. However, the anti-GQ1b antibody that is frequently associated with BBE and MFS was not detected in the patient of this study. Instead, the present case showed (equivocal) detection of anti-GD1b IgG antibody. Similarly, a case of a BBE patient was reported in whom only anti-GalNAc-GD1a IgG and anti-GalNAc-GM1b IgG antibodies were detected [31]. The patient tested positive for anti-GAD65 and anti-Ma2 antibodies, both of which have been linked to BBE [1]. As a result, BBE could not be ruled out in our patient.

Despite our patient’s atypical presentation of AE, we proceeded with immediate empiric treatment of steroids and IVIG, which was followed by PLEX. This resulted in gradual improvements. Table 1 lists the several antibodies that were examined and lists the patient's outcomes while they were in the hospital.

## Conclusions

In this case report, we have presented and discussed a rare case of AE with atypical features, with a superimposed *M. pneumoniae* infection. The patient had a past medical history of Hashimoto’s thyroiditis, PCOS, and COVID-19 infection. Upon further investigation, the patient had evidence of various subtypes of AE, namely anti-GAD65-associated antibody encephalitis, HE, BBE, and MFS. Due to the lack of standard protocol concerning the treatment of AE, we have analyzed and discussed the different treatment modalities, specifically the potential superiority of PLEX. When compared to treatment with IVIG and steroids alone, PLEX may result in significant improvement in complicated patients like the one mentioned in this article. Further research is necessary to provide a more comprehensive evidence-based treatment protocol for AE.

## Figures and Tables

**Figure 3:**
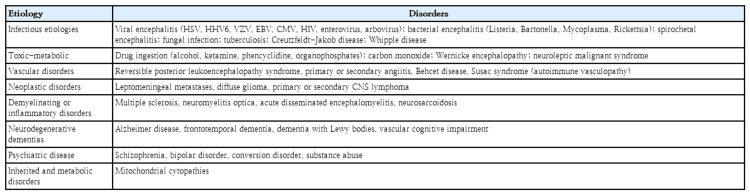
Differential diagnosis of autoimmune encephalitis Source: This image is taken from Ref. [1].
